# Involvement of peroxisome proliferator-activated receptors in cardiac and vascular remodeling in a novel minipig model of insulin resistance and atherosclerosis induced by consumption of a high-fat/cholesterol diet

**DOI:** 10.1186/s12933-014-0165-0

**Published:** 2015-01-16

**Authors:** Pan Yongming, Cai Zhaowei, Ma Yichao, Zhu Keyan, Chen Liang, Chen Fangming, Xu Xiaoping, Ma Quanxin, Chen Minli

**Affiliations:** Experimental Animal Research Center, Zhejiang Chinese Medical University, No. 548 Binwen Road, Binjiang District, Hangzhou, 310053 China

**Keywords:** Tibetan minipig, Insulin resistance, Atherosclerosis, Myocardial ischemia, PPARs, NF-ĸB, High-fat/cholesterol diet, Heart rate variability

## Abstract

**Background:**

A long-term high-fat/cholesterol (HFC) diet leads to insulin resistance (IR), which is associated with inflammation, atherosclerosis (AS), cardiac sympathovagal imbalance, and cardiac dysfunction. Peroxisome proliferator-activated receptors (PPARs) and nuclear factor ĸB (NF-κB) are involved in the development of IR-AS. Thus, we elucidated the pathological molecular mechanism of IR-AS by feeding an HFC diet to Tibetan minipigs to induce IR and AS.

**Methods:**

Male Tibetan minipigs were fed either a normal diet or an HFC diet for 24 weeks. Thereafter, the minipigs were tested for physiological and biochemical blood indices, blood pressure, cardiac function, glucose tolerance, heart rate variability (HRV), and PPAR-associated gene and protein expression levels.

**Results:**

HFC-fed minipigs exhibited IR through increased body weight, fasting blood glucose levels, plasma cholesterol and its composition, and insulin and free fatty acid (FFA) levels; decreased insulin sensitivity; impaired glucose tolerance; and hypertension. Increased C-reactive protein (CRP) levels, cardiac dysfunction, depressed HRV, and the up-regulation of PPAR expression in the abdominal aorta concomitant with down-regulation in the heart tissue were observed in HFC-fed minipigs. Furthermore, the levels of NF-κBp65, IL-1β, TNF-α, MCP-1, VCAM-1, ICAM-1, MMP-9, and CRP proteins were also significantly increased.

**Conclusions:**

These data suggest that HFC-fed Tibetan minipigs develop IR and AS and that PPARs are involved in cardiovascular remodeling and impaired function.

## Background

Hyperlipidemia is a common predicament in modern society owing to changes in lifestyle and food consumption in humans. Diet plays an important role in the management of lipoprotein and lipid concentrations in blood. Long-term, high-fat consumption leads to insulin resistance (IR), because saturated fatty acids interfere with the action of insulin [1].

IR is a precursor and the primary characteristic of type 2 diabetes. Based on an epidemiological study, the incidence of diabetes is rapidly increasing with the population aging, and approximately 9.7% of adults have diabetes in China [2]. Moreover, the mortality of heart disease is 4-fold higher in diabetes patients [3]. In fact, myocardial ischemia (MI), caused by atherosclerosis (AS) of coronary arteries, more frequently occurs without prior symptoms in these patients [4]. Therefore, the pre-diabetic pathological state of IR is concerning.

IR has been implicated in AS promotion and impaired endothelial-dependent diastolic function [5]. Data from patients with IR or elevated fasting blood glucose, increased heart rate, reduced heart rate variability (HRV) [6,7], and cardiac sympathovagal imbalance [8] and data from animal experimental and clinical studies all confirm that IR is associated with impairment of selective signaling pathways and that IR leads to peripheral vascular and myocardial structural and functional changes [9-11]. Therefore, it is necessary to establish an ideal animal model to have a better understanding of the pathological process involved in IR, hyperlipidemia, and AS, which will be helpful to further develop new therapeutic agents.

Pigs are a potentially useful animal model because, unlike mice and rabbits, their anatomy, physiology, feeding and sleeping habits, neo-intimal formation, and thrombosis are very similar to those of humans. Pigs can recapitulate the formation of diabetes- and inflammation-induced AS lesions [12,13], which are currently, mostly induced by toxicity to drugs (e.g.,streptozotocin and alloxan), causing pancreatic β-cell ablation, and AS lesions are also induced by feeding with an atherogenic diet [14,15], rather than natural development subsequent to metabolic syndrome or diabetes. In a recent study, Göttingen minipig fed a high-fat diet for 12 weeks develops obesity, IR, and lipid disorders [16], and the obesity-prone Ossabaw minipig fed a high-fat/cholesterol (HFC) diet for 9 weeks develops IR, impaired glucose tolerance, dyslipidemia, hypertension, and early coronary intimal hyperplasia [17]. However, these models do not exhibit chronic ischemia formation. Many minipig varieties in China are used in experimental studies, such as Bama minipig, WZS minipig, Guizhou minipig, and Tibetan minipig. In our previous studies using the Chinese minipig model of cardiovascular diseases, we found that HFC-fed Tibetan minipigs could also develop IR, impaired glucose tolerance, dyslipidemia, hypertension, AS, and cardiac dysfunction [18]. These conditions are very similar to IR caused by related complications in clinical cases; thus, this disease model is a novel minipig model of IR-AS and warrants further study.

Peroxisome proliferator-activated receptors (PPARs) belong to a superfamily of the type II nuclear hormone receptors, are ligand-activated transcription factors, which have three subtypes (PPAR α, β/δ, and γ), and may participate in lipid metabolism regulation and AS development [19]. PPARs also inhibit the activity of inflammation-related transcription factors (such as NF-ĸB), which inhibit inflammatory effects of a variety of inflammatory diseases. PPARs are gaining research interest with regard to metabolic and cardiovascular diseases, and *PPAR* expression in the target organ may play an important role in changing organ structure and function; moreover, PPAR subtypes function in cardiovascular diseases and may also influence lesion development. However, the specific active mechanism of PPARs in cardiovascular remodeling and dysfunction is unclear.

Therefore, we hypothesized that IR-AS with spontaneous chronic ischemia in HFC-fed Tibetan minipig maybe associated with inflammation, IR, autonomic dysfunction, and activation of PPARs and the NF-ĸB signaling pathway. In this work, we successfully recapitulated the IR-AS Tibetan minipig model induced by an HFC diet. We examined blood biochemical indices, blood pressure, cardiac function, glucose tolerance, HRV, and expression levels of genes and proteins associated with PPARs and the NF-ĸB signaling pathway. We aimed to elucidate whether HFC-fed Tibetan minipig developed IR-AS and to understand the cause of cardiac impairment and pathological molecular mechanisms. Consequently, our results provide an experimental basis for the relationship between IR and cardiovascular diseases and drug treatment.

## Methods

### Animal model

Ten male Tibetan minipigs at 3 months of age, weighing 12–14 kg, were purchased from the Experimental Animal Center of Southern Medical University, China. After adaptation to the environment for 8 weeks, Tibetan minipigs were randomly divided into 2 groups: control (Ctr) and HFC. Ctr minipigs (n = 4) were fed a diet of regular chow, and HFC minipigs (n = 6) were fed an atherogenic diet of HFC chow consisting of 1.5% cholesterol, 15% shortening oil, 10% egg yolk powder, and 73.5% regular chow (diet compositions are described in Table 1). All Tibetan minipigs in both groups received the same amount of food (2.5% of body weight) and were fed twice daily on a restricted schedule with regular chow or atherogenic diet for 24 weeks until they were euthanized. All animals were housed and fed in individual pens under a 12-h light: dark cycle. Water was provided *ad libitum*. All experiments were performed according to the guidelines proposed by the Laboratory Animal Research Center of Zhejiang Chinese Medical University (SYXK, Hangzhou, 2008-0116, China) and approved by the Institutional Animal Care and Use Committee of the Zhejiang Chinese Medical University.

**Table 1:** **Diet components**

**Component**	**Control diet**		**High-fat/high-cholesterol diet**	
	**g**	**kcal**	**g**	**kcal**
Protein	17.4	22	15.9	19
Carbohydrate	55.2	70	41.1	50
Fat	6.3	8	25.2	31
Total		100		100
kcal/g	3.39		4.46	

### Blood biochemical index measurements

At 24 weeks, blood samples were drawn from minipigs in a supine position by performing anterior vena cava puncture within 30 s after a fasting period of 12 h [20]. Blood samples were then separated to analyze total cholesterol(TC), high-density lipoprotein cholesterol (HDL-C), low-density lipoprotein cholesterol (LDL-C), triglycerides (TG), and fasting blood glucose (FBG), which were measured by an automatic biochemical analyzer (7020, HITACHI, Japan) using kits corresponding to each component (Shanghai Shenneng-DiaSys Diagnostic Technology Co., Ltd., China) for each test. The serum free fatty acid (FFA), insulin, and C-reactive protein (CRP) levels were measured using homologous specific ELISA kits (Shanghai Xinran Biotech Co, Ltd., China) according to the manufacturer’s instructions. The homeostasis model assessment IR (HOMA-IR) method was used to evaluate insulin resistance. HOMA-IR was calculated using the following formula: FBG × insulin/22.5 [21].

### Intravenous glucose tolerance test (IVGTT)

The IVGTT was performed in each minipig after 8 and 24 weeks of assigned diet consumption. Minipigs were acclimated to restraint in a specialized fixed sling for 3 days before IVGTT. They were anesthetized with isoflurane (maintained at 2–3% by mask with supplemental O_2_), and then right jugular veins were catheterized percutaneously [22]. Minipigs were allowed to recover for 3 h before IVGTT to avoid any effects of isoflurane on glucose levels [23]. Blood samples were obtained at the baseline time point (0 min), then glucose (0.5 g/kg) was injected intravenously over 2 min, and further samples were obtained at 15, 30, 60, 90, and 120 min after injection for measurement of blood glucose and insulin concentrations. From the IVGTT, the insulin sensitivity index (S2) for minipigs was calculated as previously described [24].

### Blood pressure and cardiac function measurement

Minipigs were acclimated to a low-stress sling for blood pressure measurements. Non-invasive blood pressure was monitored using a non-invasive physiological signal telemetry system (EMKA Technologies S.A.S., France) by using a tail cuff method at the beginning of the experiment and 12 weeks into the experiment. Mean blood pressure (MBP), systolic blood pressure (SBP), and diastolic blood pressure (DBP) were analyzed by ECGAUTO software (EMKA Technologies). After 24 weeks, all minipigs were anesthetized with 10 mg/kg of intramuscular xylazine hydrochloride (Huamu Animal Health Products Co., Ltd., Jilin, China) and intubated, and anesthesia was maintained by inhalation of 0.5–2% isoflurane combined with oxygen and room air [25]. During anesthesis, minipigs were placed on heating pads maintained at 37°C, and the core temperature was measured via a rectal probe (Life Window 6000; Digicare Animal Health, Boynton Beach, FL, USA). All minipigs were equipped with two polyethylene blood vessel catheters. One catheter was inserted into the left ventricle via the right common carotid artery to record cardiac function parameters, and another catheter was inserted into the left femoral artery to record blood pressure parameters. After stabilizing for 20 min (i.e., after intramuscular injection of xylazine hydrochloride) at 80 min, when the effects of xylazine hydrochloride on blood pressure are negligible [26], the signals were recorded continuously by using a non-invasive physiological signal telemetry system (EMKA Technologies). MBP, SBP, DBP, left ventricular systolic pressure (LVSP), left ventricular end diastolic pressure (LVEDP), and the maximum rate of left ventricular pressure rise and fall (±dp/dt_max_) were analyzed by ECGAUTO software (EMKA Technologies).

### HRV analysis

The physiological telemetry test was performed for each minipig every 8 weeks. All minipigs were acclimatized to the experimental jackets for 3 days before each test. Minipigs were shaved, and surface ECG electrodes were attached. Then, specially designed jackets were placed on each minipig to protect the ECG electrodes and lead wires, and minipigs were returned to their cages. After stabilization for 2 h, vital signs of each animal (Lead I and II ECG) were monitored for 24 h and recorded using a Non-Invasive Physiological Signal Telemetry System (EMKA Technologies). ECG signals were recorded at a sampling rate of 500 Hz by EMKA IOX data acquisition software. ECG-AUTO software (EMKA Technologies) was used for ECG, activity, and HRV analyses. Data from the 24 h sampling period were divided into 30-minsegments of RR data. R waves were detected, and the RR interval (RRI) tachogram was calculated as raw HRV in sequence order. Similarly, from the same recordings, HRV was analyzed for time and frequency domains. The time-domain parameters obtained were as follows: mean of normal RRIs, standard deviation of all normal RRIs (SDNN), and square root of the mean square successive differences between successive normal intervals (rMSSD). From this tachogram, data sets of 1024 points were re-sampled at 286 ms. A Hamming window was applied to each data set to minimize spectral leakage. A fast Fourier transform was used to obtain the power spectrum of the fluctuation. We identified frequency bands of very low frequency (VLF; 0.003–0.01 Hz), low frequency (LF; 0.01–0.07 Hz), and high frequency (HF; 0.07–1.0 Hz) according to a previous study [27]; the sympathetic and vagal modulation ratio balance was defined by the LF/HF ratio.

### Body composition

The percentage of carcass fat was calculated according to the following equation: carcass fat weight/body weight × 100%. Body mass index was measured as in humans [28] and was calculated by the following equation: (body weight in kg)/(square of the minipig length from the end of the snout to the base of the tail).

### Myocardium ischemia size (MIS) determination

MIS was assessed by nitrotetrazolium blue chloride (NBT; Sinopharm Chemical Reagent Co., Ltd., China) staining, as described previously [29]. Briefly, at 24 weeks, the heart was excised, washed in chilled saline, weighed immediately, and then dissected to remove the atria and right ventricle. The left ventricle was frozen at − 20°C, cut in a semi-frozen state into 5 equally thick sections, and incubated in 0.1% NBT solution for 15 min at 37°C. The non-ischemia area stained blue, whereas the ischemia area was not stained. The area of ischemia was measured in each slice with Image J software (version 1.4, National Institutes of Health, USA) and was expressed as the percentage of the total left ventricular area.

### Pathological examination

Upon anesthesis at 24 weeks, the chest of each minipig was opened to achieve euthanasia. Samples of the aorta, arteries, heart, pancreas, liver, and adipose tissues were removed from each animal. The thoracic and abdominal portions of the aortas were longitudinally incised and fixed with 10% buffered formalin for 24 h, followed by staining with Oil Red O stain for lipid deposition analysis. The Oil Red O-stained area relative to the entire surface was calculated using Image J software. The heart, pancreas, abdominal aorta, and left branch of the coronary artery were fixed overnight with 10% phosphate-buffered formalin, dehydrated, embedded in paraffin, sectioned into 5-μm-thick slices, and stained with hematoxylin and eosin (H&E). The intima-media thickness (IMT) was measured at the point of greatest thickness in sections from the standardized sample sites by using Image J software.

### RNA extractions and quantitative real-time PCR (qPCR)

Total RNA was extracted from the abdominal aorta and myocardium tissue using TAKARA RNAiso Plus Reagent according to the instructions. For each sample, first-strand cDNA was synthesized using the Prime Script RT Reagent Kit (TaKaRa, Dalian, China). The sequences of the primers were as follows: porcine *GAPDH* sense, 5′-AGGTCGGAGTGAACGGATTTG-3′, *GAPDH* anti-sense 5′-ACCATGTAGTGGAGGTCAATGAAG-3′; *PPAR-α* sense, 5′-GCTGCTCAGCACCAAGGT-3′, *PPAR-α* anti-sense, 5′-ACCTCCTTCATGCTCTCCTCTAT-3′; *PPAR-β/δ* sense 5′-CTACAAGTGCCAGTGTGAGGAG-3′, *PPAR-β/δ* anti-sense 5′-GGTTGGTGAAGAAGAGGTAGGC-3′; and *PPAR-γ* sense 5′-GACGCAGATTTTGTAGACG-3′, *PPAR-γ* anti-sense 5′-CCCTACTGGTTTCTGGATG-3′. The expected sizes of synthesized cDNA were 125 bp for *GAPDH*, 115 bp for *PPAR-α*, 95 bp for *PPAR-β/δ*, and 81 bp for *PPAR-γ*. qPCR was performed in an automated thermal cycler (Minioption™ Real-time PCR System; BIO-RAD) in a final volume of 25 μL, containing 2 μL of cDNA solution, 12.5 μL of SYBR® Premix Ex Taq (TaKaRa, Dalian, China), 1 μL of each primer (10 μmol/L), and 8.5 μL of dH_2_O. The cycling reaction was performed according to the manufacturer’s instructions via a standard two-step PCR. *GAPDH* was used as a housekeeping gene to normalize target gene expression, and the expression levels were determined by the comparative CT method.

### Abdominal aorta and myocardial protein extraction and western blotting analysis

Abdominal aorta and myocardial proteins were extracted according to the method provided by the manufacturer of the extraction kit (Key Gen Bio TECH, Nanjing, China).The concentrations of total protein in the supernatant were determined by the BCA method, and then 2% sodium dodecyl sulfate (SDS) and 5% 2-mercaptoethanol were added to denature proteins at 100°C for 10 min. Fifty micrograms of protein was separated by SDS-polyacrylamide gel electrophoresis and transferred to polyvinylidenedifluoride membranes. The membranes were blocked with 5% non-fat milk in 20 mmol/L Tris-buffered saline (pH 7.4) containing 500 mmol/L NaCl and 0.05% Tween 20 (TBST) and then incubated with primary antibody (1:150 dilution, PPAR-α, SC-1982; PPAR-γ, SC-1981; and GAPDH, SC-166545: Santa Cruz Biotechnology, USA; PPAR-β/δ, ab23673: Abcam, USA) overnight at 4°C. Thereafter, the membranes were washed three times with TBST and incubated with a 1:10,000 dilution of horseradish peroxidase-conjugated secondary antibody for 1 h at room temperature. Membranes were again washed three times in TBST. Finally, signals were detected using the Odyssey Infrared Fluorescent Scanner (LI-COR, USA) and quantified by Image J software.

### Immunohistochemistry

Fresh abdominal aortas were sliced into 7-μm-thick frozen sections, blocked with 3% hydrogen peroxide solution for 10 min to eliminate the activity of endogenous peroxidase, and then incubated overnight at 4°C in the following polyclonal antibody solutions: anti-NF-ĸB p65, anti-interleukin 1 beta (IL-1β), anti-tumor necrosis factor alpha (TNF-α), anti-monocyte chemoattractant protein-1 (MCP-1), anti-vascular cell adhesion molecule 1 (VCAM-1), anti-intercellular adhesion molecule 1 (ICAM-1), anti-matrix metalloproteinase 9 (MMP-9), and anti-CRP (Zhongshan GoldenBridge, Beijing, China). Subsequently, samples were washed with phosphate- buffered saline three times, and the sections were incubated with secondary antibodies for 1 h. After incubation, the sections were stained using 3, 3′-diaminobenzidine tetrahydrochloride (DAB) as a color reagent and counterstained with hematoxylin. The stained slides were viewed with a light microscope (Nikon Eclipse 80i), and digital images of six randomly selected, high-power (×400 magnification) fields were captured on NIS- ElementS.F.2.30 software (Nikon United Kingdom Ltd., Surrey, UK). Protein expression was quantified with Image Pro Plus 5.0 software. This software discriminates between positive- and negative-stained pixels. A visual check was also performed to ensure accurate discrimination of immunolabeled regions. Positive expression is represented as the percentage of the total area.

### Statistical analysis

Data are expressed as means ± SEM. SPSS 17.0 software (SPSS, Chicago, IL, USA) was used to perform statistical analysis. Data were compared using unpaired student’s t tests. A two-tailed *P* value <0.05 was considered statistically significant.

## Results

### General characteristics of Ctr and HFC-fed Tibetan minipigs

**Figure 1:**
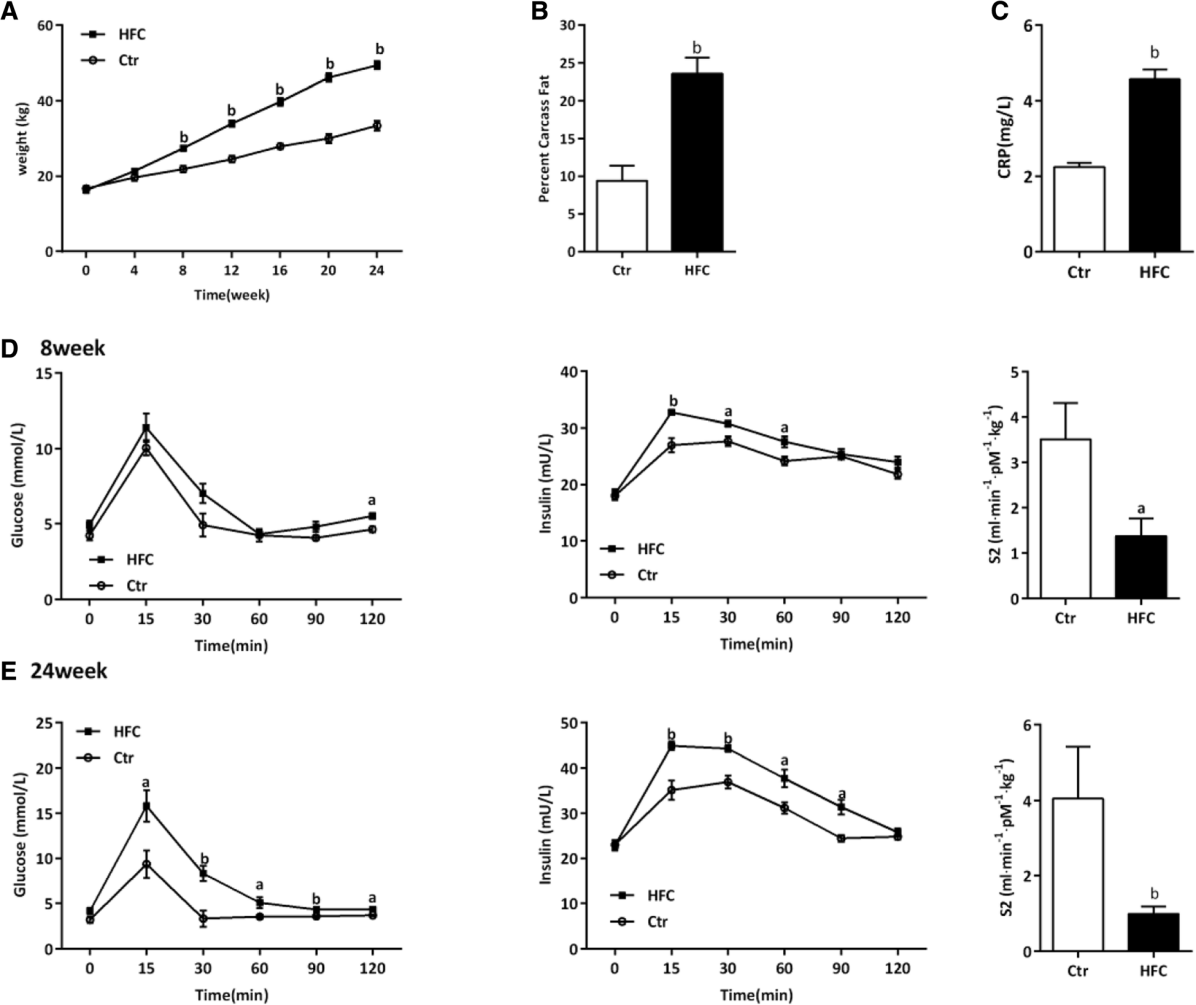
**Weight, Body fat, CRP, and intravenous glucose-intolerant test (IVGTT) results in Tibetan minipigs.** Control (Ctr, n = 4) and high-fat/cholesterol atherogenic diet minipigs (HFC, n = 6). **(A)** Weight, **(B)** percentage of carcass fat, **(C)** C reaction protein (CRP), **(D)** simultaneous measurement of glucose and insulin responses during IVGTT and insulin sensitivity index (S2) at 8 week, **(E)** simultaneous measurement of glucose and insulin responses during IVGTT and S2 at 24 week. Data are presented as mean ± SEM. ^a^, *P* < 0.05 and ^b^, *P* < 0.01 versus Ctr.

**Figure 2:**
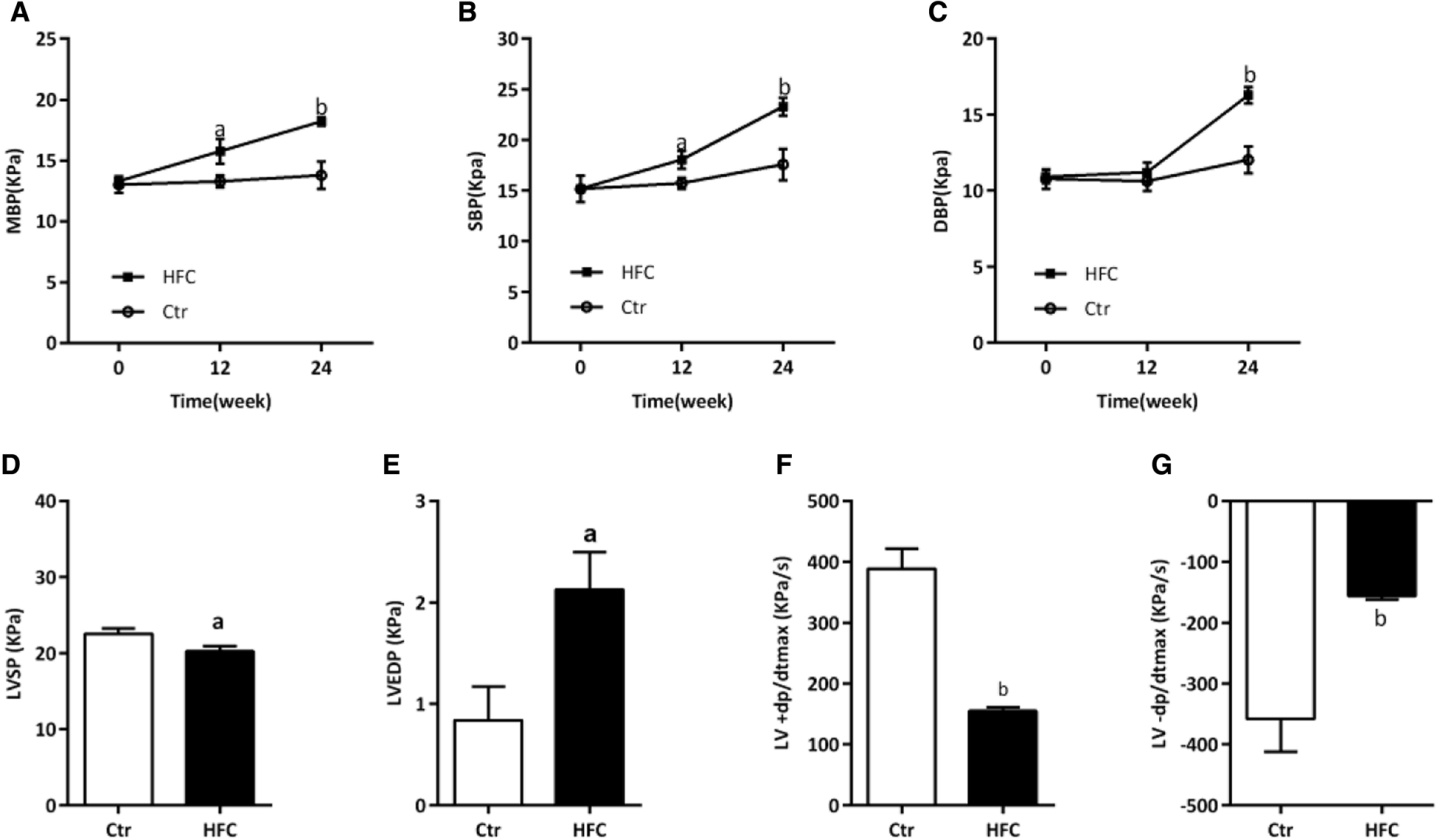
**Blood pressure and cardiac hemodynamic parameters in Tibetan minipigs.** Control (Ctr, n = 4) and high-fat/cholesterol atherogenic diet minipigs (HFC, n = 6). **(A)** Mean blood pressure (MBP), **(B)** systolic blood pressure (SBP), **(C)** diastolic blood pressure (DBP), **(D)** left ventricular systolic pressure (LVSP), **(E)** left ventricular end diastolic pressure (LVEDP), **(F)** LV + dp/dt_max_, and **(G)** LV –dp/dt_max_. Data are presented as mean ± SEM. ^a^, *P* < 0.05 and ^b^, *P* < 0.01 versus Ctr.

Tibetan minipigs were fed an HFC diet for 24 weeks to induce IR-AS. HFC-fed minipigs had significantly increased body weight, body mass, TC, HDL-C, LDL-C, FFA, FBG, insulin, and HOMA-IR index compared with Ctr minipigs (*P* < 0.01, Table 2), and the TG level also showed a trend of increasing (*P* > 0.05, Table 2). Carcass fat and CRP level of HFC-fed minipigs were significantly higher than those of Ctr-fed minipigs (*P* < 0.01, Figure 1B and C). The IVGTT showed that HFC-fed minipigs had significantly higher blood glucose and insulin levels during the 120 min after 0.5 mg/kg intravenous injection of glucose at 8 or 24 weeks, and the S2 was also decreased significantly (*P* < 0.05, *P* < 0.01, Figure 1D and E). In addition, HFC-fed minipigs showed significantly elevated MBP and SBP at 12 or 24 weeks (*P* < 0.05, *P* < 0.01, Figure 2A and B) and elevated DBP at 24 weeks (*P* < 0.01, Figure 2C). Cardiac hemodynamic results showed that HFC-fed minipigs had significantly lower LVSP and LV ± dp/dt_max_ but significantly higher LVEDP than Ctr minipigs (*P* < 0.05, *P* < 0.01, Figure 2D–G).

**Table 2:** **Phenotypic characteristics of Tibetan minipigs fed a control (Ctr) or high-fat/cholesterol atherogenic (HFC) diet**

**Parameters**	**Ctr group**	**HFC group**	***P-*** **value**
Starting body weight (kg)	16.73 ± 0.72	16.30 ± 0.54	NS
End body weight (kg)	33.43 ± 1.29	49.40 ± 1.07^b^	<0.0001
End body mass index (kg/m^2^)	41.98 ± 1.35	57.05 ± 1.93^b^	0.0002
Total cholesterol (mg/dL)	96.08 ± 7.96	406.60 ± 42.26^b^	0.0006
HDL-C (mg/dL)	33.77 ± 3.42	120.60 ± 5.34^b^	<0.0001
LDL-C (mg/dL)	42.76 ± 5.55	186.30 ± 21.93^b^	0.0009
Triglycerides (mg/dL)	23.61 ± 2.31	25.38 ± 3.69	NS
Free fatty acids (nmol/L)	0.51 ± 0.01	0.61 ± 0.01^b^	0.0002
Fasting blood glucose (mmol/L)	4.64 ± 0.10	5.47 ± 0.20^b^	0.0076
Fasting insulin (mU/L)	18.05 ± 0.74	21.61 ± 0.49^b^	0.0083
HOMA-IR	3.71 ± 0.09	5.24 ± 0.17^b^	<0.0001

Ctr: control group animals (n = 4); HFC: high-fat/cholesterol atherogenic diet animals (n = 6); HDL-C: high-density lipoprotein cholesterol; LDL-C: low-density lipoprotein cholesterol; HOMA-IR: homeostasis model assessment of insulin resistance. Values are shown as mean ± SEM. ^a^, *P* < 0.05 and ^b^, *P* < 0.01 versus control group. NS: not significant.

### HRV analysis

**Figure 3:**
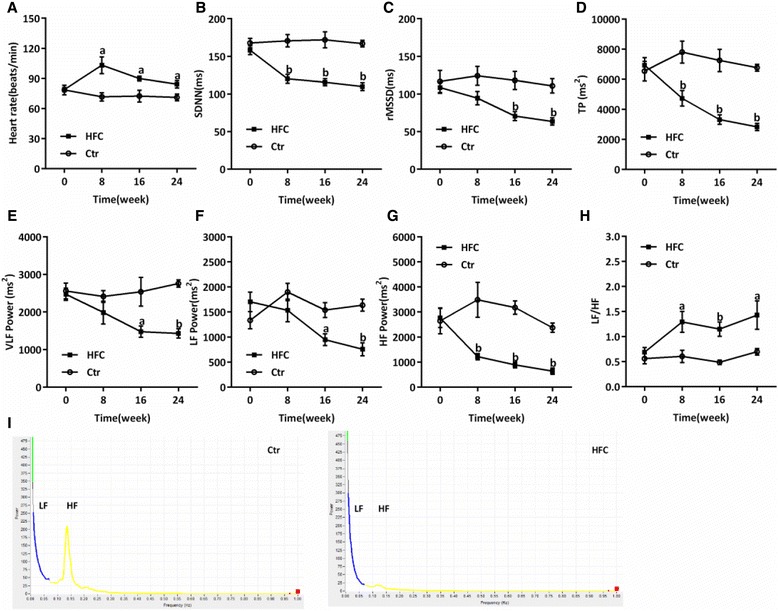
**Heart rate variability analysis in Tibetan minipig.** Control (Ctr, n = 4) and high-fat/cholesterol atherogenic diet minipigs (HFC, n = 6). **(A)** Heart rate, **(B)** standard deviation of all normal RR intervals (SDNN), **(C)** square root of the mean square successive differences between successive normal intervals (rMSSD), **(D)** total power (TP), **(E)** very low frequency (VLF) power, **(F)** low frequency (LF) power, **(G)** high frequency (HF) power, **(H)** LF/HF ratio, **(I)** graph of frequency domain analysis. Data are presented as mean ± SEM. ^a^, *P* < 0.05 and ^b^, *P* < 0.01 versus Ctr.

HFC-fed minipigs exhibited significantly reduced SDNN, total power (TP), and HF power at 8 weeks after experiment initiation compared with the Ctr group (*P* < 0.05, *P* < 0.01, Figure 3B,D, and G). rMSSD and VLF and LF power levels were also significantly reduced at 16 weeks (*P* < 0.05, *P* < 0.01, Figure 3C,E, and F); however, the heart rate and LF/HF ratio were significantly high at 8 weeks (*P* < 0.05, *P* < 0.01, Figure 3A and H). In addition, the frequency domain analysis also showed that the LF and HF power levels were reduced in the HFC group compared with the Ctr group (Figure 3I).

### Histopathological assessment

**Figure 4:**
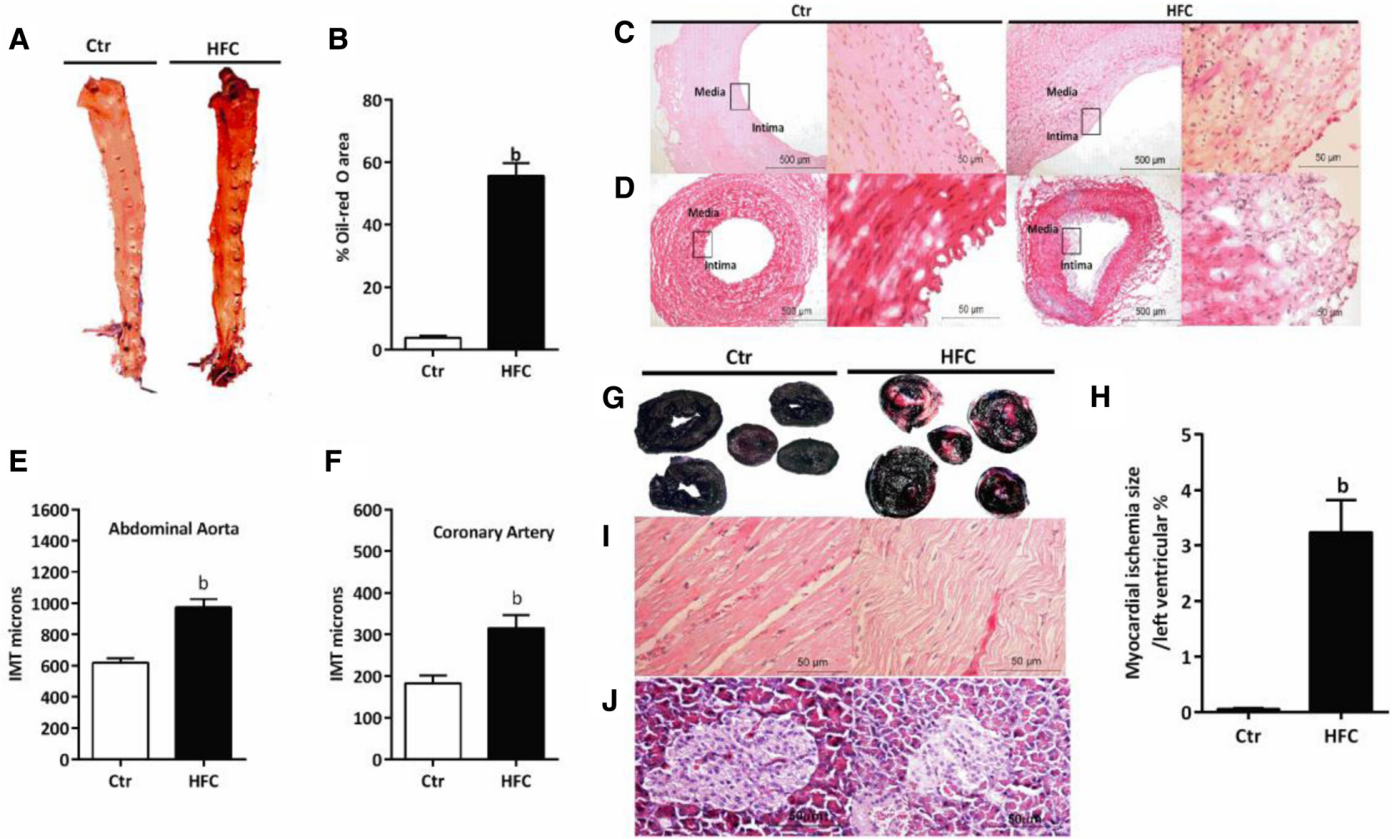
**Histopathological assessment in Tibetan minipigs.** Control (Ctr, n = 4) and high-fat/cholesterol atherogenic diet minipigs (HFC, n = 6). **(A)** Aortic lipid analysis by Oil Red O staining, **(B)** percent of Oil Red O area, **(C)** H&E-stained abdominal aorta (left is H&E 40×, right is H&E 400×), **(D)** HE-stained coronary artery (left is H&E 40×, right is H&E 400×), **(E)** IMT of abdominal aorta, **(F)** IMT of coronary artery, **(G)** myocardial tissue with NBT staining, **(H)** myocardial ischemia size/left ventricular, **(I)** H&E-stained myocardial tissue (H&E 400×), **(J)** H&E-stained pancreatic tissue (H&E 400×). Data are presented as mean ± SEM. ^b^, *P* < 0.01 versus Ctr.

Oil Red O staining analysis demonstrated that aortic atherosclerotic lesions significantly increased in the HFC group compared with the Ctr group (*P* < 0.01, Figure 4A and B), and we also observed that the atherosclerotic lesions developed first at the aortic arch and abdominal aorta and progressed to involve the entire aorta in the HFC group. H&E staining showed that abdominal aorta and coronary artery atherosclerotic lesions were mainly located in the intima. The lesions were composed of foam cell infiltration, thickening of the intima, and protrusion into the lumen, and the elastic membrane of the plaque bottom was destroyed with infiltration of inflammatory cells (Figure 4C and D). The IMTs of the abdominal aorta and coronary artery were significantly increased in the HFC group compared to the Ctr group (*P* < 0.01, Figure 4E and F).These data strongly suggest that HFC-fed minipigs can form obvious atherosclerotic lesions. In addition, we were surprised to find that HFC-fed minipigs experienced spontaneous myocardial infarction and islet cell changes. NBT staining demonstrated that MIS was significantly increased in the HFC group compared with the Ctr group (*P* < 0.01, Figure 4G and H). H&E staining further showed that HFC-fed minipigs had disorderly arranged myocardial fibers with a wave-like appearance, narrowed muscle gaps, and hypertrophic cardiomyocytes, while no changes were observed in the Ctr group (Figure 4I). Moreover, partial islet cell ablation and mild cloudy swelling were observed in the HFC group, but no changes were observed in the Ctr group (Figure 4J).

### Up-regulation of PPAR gene and protein expression in the abdominal aorta of HFC-fed Tibetan minipigs

**Figure 5:**
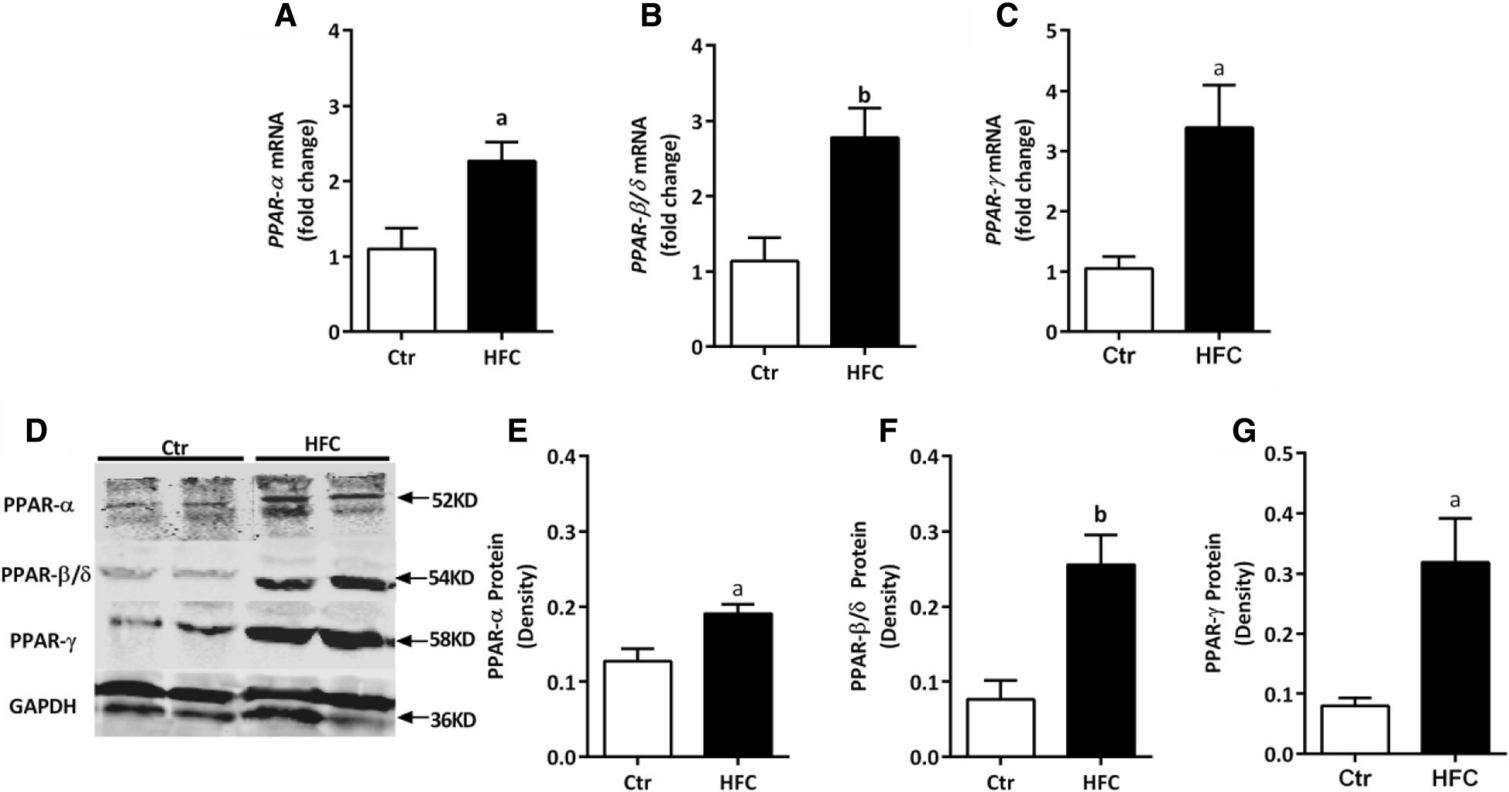
**Gene expression and protein levels in the abdominal aorta tissue in Tibetan minipigs.** Gene expression was measured by qPCR, and protein levels were measured by western blot assay in control (Ctr) and high-fat/cholesterol atherogenic diet minipigs (HFC). **(A)** *PPAR-α* mRNA, **(B)** *PPAR-β/δ* mRNA, **(C)** *PPAR-γ* mRNA, **(D)** western blot of PPAR proteins, and quantification of **(E)** PPAR-α, **(F)** PPAR-β/δ, and **(G)** PPAR-γ proteins. Data are presented as mean ± SEM. ^a^, *P* < 0.05 and ^b^, *P* < 0.01 versus Ctr group.

PPAR gene and protein expression levels in the abdominal aorta were assessed to confirm whether activated PPARs participate in an important pathological mechanism in the Tibetan minipig model of IR-AS. Compared with the Ctr group, HFC-fed minipigs had significantly higher levels of *PPAR-α*, *PPAR-β/δ*, and *PPAR-γ* mRNAs and corresponding proteins in abdominal aortic tissues (*P* < 0.05, *P* < 0.01,Figure 5A–G).

### Down-regulation of PPAR gene and protein expression in the heart of HFC-fed Tibetan minipigs

**Figure 6:**
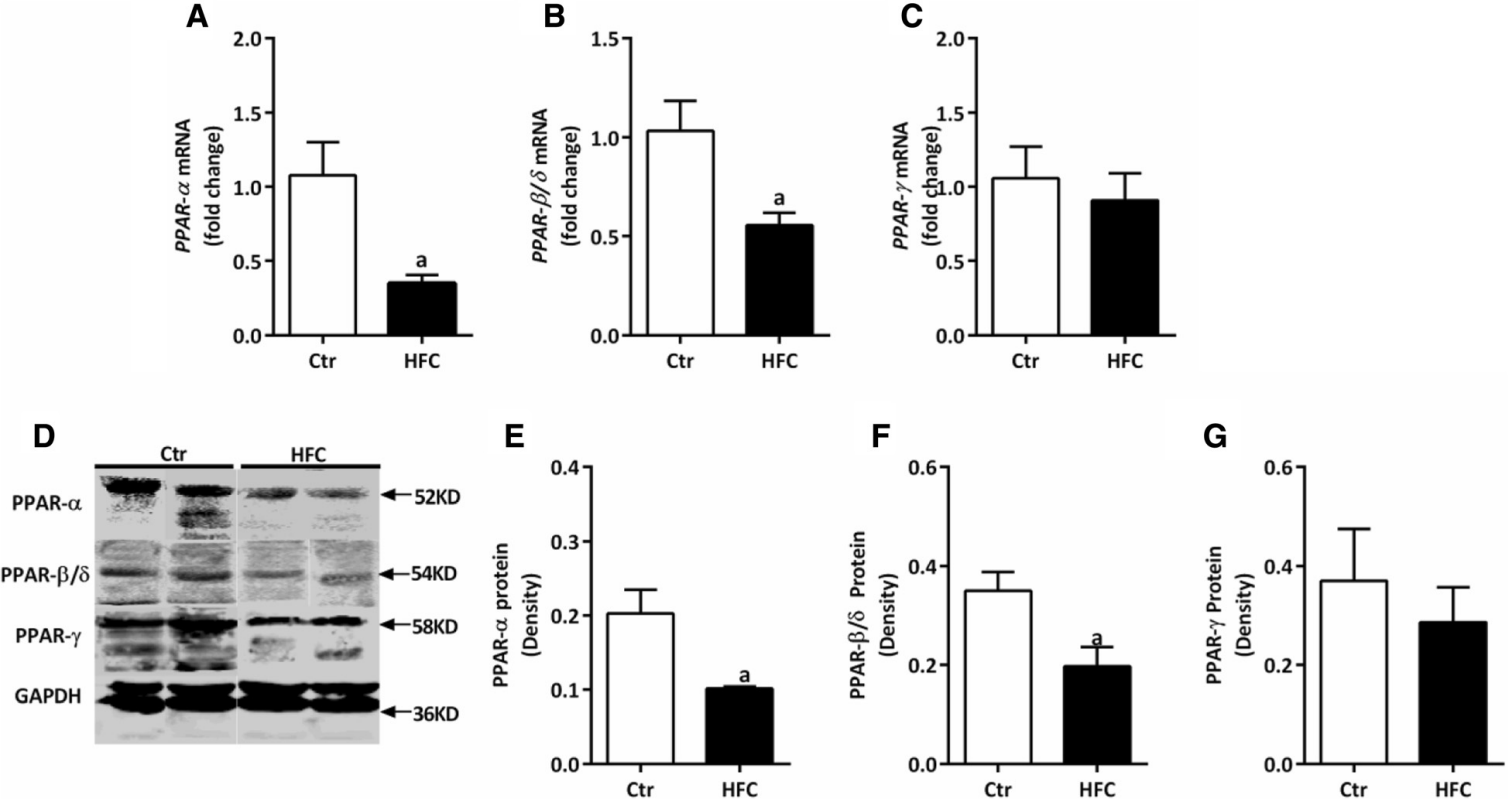
**Gene expression and protein levels in the myocardial tissue of Tibetan minipigs.** Gene expression was measured by qPCR, and protein levels were measured by western blot assay in control (Ctr) and high-fat/cholesterol atherogenic diet minipigs (HFC). **(A)** *PPAR-α* mRNA, **(B)** *PPAR-β/δ* mRNA, **(C)** *PPAR-γ* mRNA, **(D)** western blot of PPAR proteins, and quantification of **(E)** PPAR-α, **(F)** PPAR-β/δ, and **(G)** PPAR-γ proteins. Data are presented as mean ± SEM. ^a^, *P* < 0.05 versus Ctr.

qPCR and western blotting showed that mRNA and protein levels, respectively, of PPAR-α and PPAR-β/δ were significantly reduced in the heart tissue of HFC-fed minipigs (*P* < 0.05, Figure 6A–B, D–F).

### NF-κB pathway activation and up-regulation of inflammatory cytokines in the abdominal aorta of HFC-fed Tibetan minipigs

**Figure 7:**
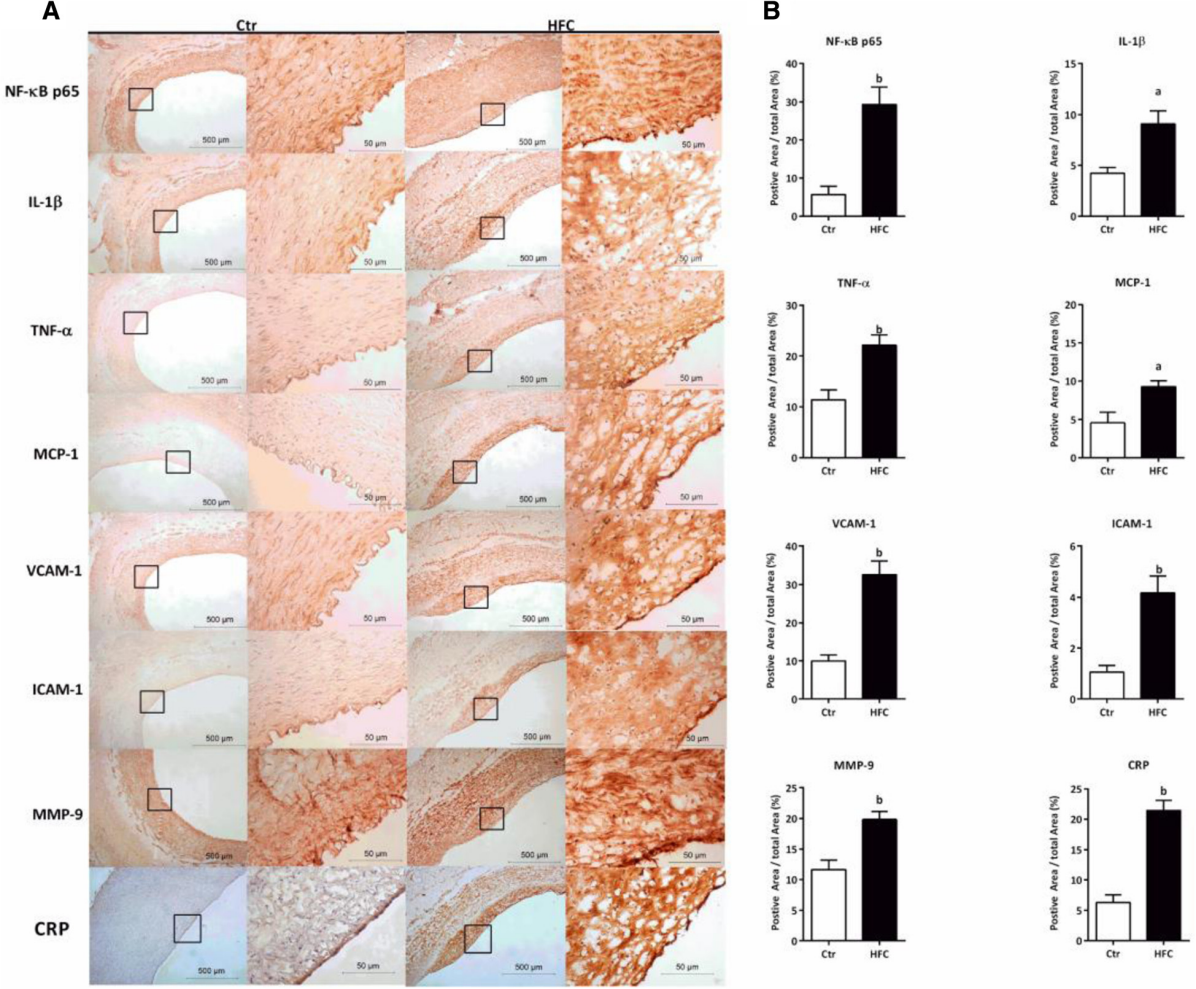
**Immunohistochemical staining in the abdominal aorta of control (Ctr) and high-fat/cholesterol atherogenic diet minipigs (HFC). (A)** Immunohistochemical staining for inflammatory cytokines in the abdominal aorta, NF-ĸB p65: nuclear factor ĸB p65; IL-1β: interleukin 1 beta; TNF-α: tumor necrosis factor alpha; MCP-1: monocyte chemoattractant protein-1; VCAM-1: vascular cell adhesion molecule 1; ICAM-1: intercellular adhesion molecule 1; MMP-9: matrix metalloproteinase 9; CRP: C-reactive protein; left, 40×; and right, 400×. **(B)** NF-ĸB p65, IL-1β, TNF-α, MCP-1, VCAM-1, ICAM-1, MMP-9, and CRP protein levels were quantified in HFC and Ctr samples. Data are presented as mean ± SEM. ^a^, *P* < 0.05 and ^b^, *P* < 0.01 versus the Ctr group.

NF-ĸB protein levels increased in the HFC group compared with the Ctr group (Figure 7A and B). Moreover, immunohistochemical analysis showed that positive staining for IL-1β, TNF-α, MCP-1, VCAM-1, ICAM-1, MMP-9, and CRP proteins significantly increased in the HFC group compared with the Ctr group (*P* < 0.05, *P* < 0.01, Figure 7B), and staining was primarily located in the endothelium of intimal lesions and within the fibrous lesions (Figure 7A).

## Discussion

In the current study, we aimed to establish a Tibetan minipig model of IR-AS by feeding an HFC diet, which resulted in significant weight gain, dyslipidemia, glucose intolerance, hypertension, inflammation, and activated the PPARs and NF-ĸB signaling pathways. These abnormalities are known to develop in IR and AS; thus, the Tibetan minipig model may be helpful to further understanding of human IR and cardiovascular disease development.

Other investigators have induced IR in pig models by using high-energy diets enriched with cholesterol and fat, but IR reportedly develops at different times, e.g., 2 weeks [30] and 16 weeks [31,32], which may be related to composition of diet and pig strains. A recent report stated that the IVGTT-derived S2 is more sensitive to evaluate insulin sensitivity, which has been shown to correlate with clamp insulin sensitivity in pigs [30]. Interestingly, we also found that 8 weeks of feeding an HFC diet were sufficient to induce glucose intolerance and IR in Tibetan minipigs.

Additionally, HFC-fed Tibetan minipigs had significantly increased plasma FFA concentration and blood pressure and depressed HRV with increased sympathetic activities. Wang et al. [33] also reported that a high-fat/ high-carbohydrate diet fed to pigs could induce artery IR, vascular dysfunction, and hypertension. Accumulating evidence links elevated plasma FFA with IR, hypertension [34,35], and activation of the sympathetic nervous system and aggravates MI [36]. FFAs are regarded as potential biochemical markers of post-infarct myocardial remodeling [37]. Further, IR is also associated with left ventricular remodeling [38] and often is accompanied by hemodynamic stress, such as systemic hypertension, which could cause left ventricular pressure overload and result in heart failure [11]. Therefore, a long term HFC diet increases plasma FFA and aggravates IR, which leads to severe cardiac autonomic nervous dysfunction. Specifically, the interaction between the sympathetic nervous system and inflammation plays an important role in the development of vascular stiffening and AS [39]. Moreover, increased FFA was associated with augmented myocardial oxygen consumption [40], limited myocardial blood supply, and cardiac dysfunction, all of which further promote chronic MI in HFC-fed minipigs.

The most interesting, and possibly still largely underestimated, role of FFAs in cell signaling is their ability to bind to nuclear receptors such as PPARs. PPAR-α, PPAR-β/δ, and PPAR-γ are the ligand-activated transcription factors that function as the master regulators of insulin sensitivity, glucose homeostasis, fatty acid and lipoprotein metabolism, inflammation, and AS [41-47]. Considering this function, any dysregulation of these metabolic pathways may result in obesity, diabetes, and cardiovascular diseases. Previous animal experiments have identified that *PPAR-α* or *PPAR-β/δ* deficiency could reduce myocardial fatty acid oxidation, increase glucose utilization and myocardial hypertrophy, and increase ischemic heart sensitivity [42,48]. Similar to *PPAR-α* and *PPAR-β/δ* deficiency, *PPAR-γ*-defective mice also exhibit severe myocardial hypertrophy [43,44]. Interestingly, PPAR-α and PPAR-β/δ expression in heart tissue of HFC-fed minipigs is significantly down-regulated (Figure 6). Thus, we speculate that PPAR-α and PPAR-β/δ may have dominant roles in regulating myocardial fatty acid metabolism, instead of PPAR-γ; further, PPAR-γ may use an indirect mechanism to achieve cardioprotective effects, probably by mobilizing fat metabolism and directing glucose and fatty acid transport to the heart.

**Figure 8:**
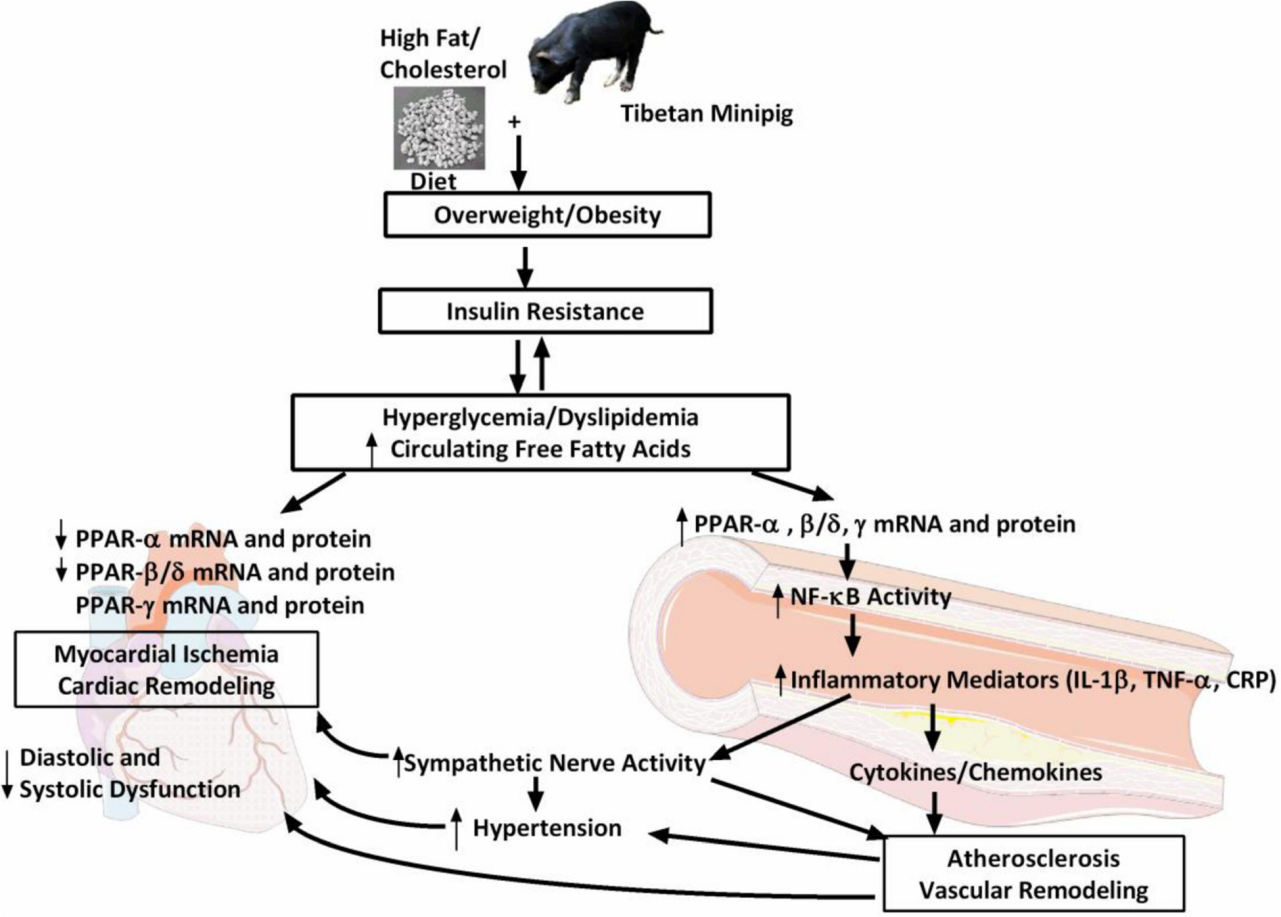
**Schematic representation of insulin resistance and atherosclerosis mechanisms in Tibetan minipig.** Conditions were induced by feeding with a high-fat/cholesterol diet in minipigs with chronic myocardial ischemia. PPARs, peroxisome proliferator-activated receptors; NF-ĸB, nuclear factor ĸB; IL-1β, interleukin 1 beta; and TNF-α: tumor necrosis factor alpha; CRP, C-reactive protein. Arrows pointing up or down indicate statistically significant increases or decreases (*P* < 0.05). Lack of arrows indicates no alteration. The heart and vessel figures were prepared using a template on the Servier Medical Art website (http:// www.servier.fr/servier-medical-art).

Moreover, *PPAR* genes are also expressed in vascular smooth muscle cells (VSMCs), endothelial cells, and monocytes/macrophages [45,46]. PPARs are highly expressed during monocyte to macrophage differentiation [47]. Some evidence implicates *PPAR-α* and *PPAR-γ* in atherogenesis and vascular remodeling of hypertension [47,49,50]. *PPAR-β/δ* is also expressed in VSMCs and up-regulated after vascular injury [51]. Meanwhile, PPARs can also repress gene expression by antagonizing the activities of NF-κB and activator proteins [46]. Our results also demonstrated that *PPAR* gene expression and protein levels were both up-regulated in AS lesions of the abdominal aorta, which is consistent with the results of previous clinical studies by Shchelkunova et al. [52]. Additionally, NF-κB protein and levels of its downstream inflammatory factors, such as IL-1β, TNF-α, ICAM-1, VCAM-1, MCP-1, MMP-9, and CRP were also increased in HFC-fed minipigs (Figure 8). Thus, HFC-fed minipigs show obvious vascular remodeling and impaired function in the aorta. Therefore, it is clear that the PPAR and NF-κB signaling pathways are involved in the development of IR-AS in HFC-fed minipigs.

## Conclusions

Our findings describe a novel minipig model where by feeding Tibetan minipigs an HFC diet produced IR-AS, including dyslipidemia, IR, glucose intolerance, hypertension, and inflammatory and vascular AS lesions. PPARs are involved in cardiovascular remodeling and impaired function. We propose that this Tibetan minipig model may be helpful to further understanding of human IR and cardiovascular disease development.

## References

[1] Funaki M (2009). Saturated fatty acids and insulin resistance. J Med Invest.

[2] Deng W, Zhang Y, Zheng Y, Jiang Y, Wu Q, Liang Z (2014). Serum retinol-binding protein 4 levels are elevated but do not contribute to insulin resistance in newly diagnosed Chinese hypertensive patients. Diabetol Metab Syndr.

[3] Ye Y, Perez-Polo JR, Aguilar D, Birnbaum Y (2011). The potential effects of anti-diabetic medications on myocardial ischemia-reperfusion injury. Basic Res Cardiol.

[4] Alatab S, Fakhrzadeh H, Sharifi F, Mostashfi A, Mirarefin M, Badamchizadeh Z (2014). Impact of hypertension on various markers of subclinical atherosclerosis in early type 2 diabetes. J Diabetes Metab Disord.

[5] Kearney MT, Duncan ER, Kahn M, Wheatcroft SB (2008). Insulin resistance and endothelial cell dysfunction: studies in mammalian models. Exp Physiol.

[6] Stein PK, Barzilay JI, Domitrovich PP, Chaves PM, Gottdiener JS, Heckbert SR (2007). The relationship of heart rate and heart rate variability to non-diabetic fasting glucose levels and the metabolic syndrome: the Cardiovascular Health Study. Diabet Med.

[7] Stein PK, Barzilay JI, Chaves PH, Traber J, Domitrovich PP, Heckbert SR (2008). Higher levels of inflammation factors and greater insulin resistance are independently associated with higher heart rate and lower heart rate variability in normoglycemic older individuals: the Cardiovascular Health Study. J Am Geriatr Soc.

[8] Apaijai N, Pintana H, Chattipakorn SC, Chattipakorn N (2013). Effects of vildagliptin versus sitagliptin, on cardiac function, heart rate variability and mitochondrial function in obese insulin-resistant rats. Br J Pharmacol.

[9] Muniyappa R, Iantorno M, Quon MJ (2008). An integrated view of insulin resistance and endothelial dysfunction. Endocrinol Metab Clin North Am.

[10] Deng JY, Huang JP, Lu LS, Hung LM (2007). Impairment of cardiac insulin signaling and myocardial contractile performance in high-cholesterol/fructose-fed rats. Am J Physiol Heart Circ Physiol.

[11] Raher MJ, Thibault HB, Buys ES, Kuruppu D, Shimizu N, Brownell AL (2008). A short duration of high-fat diet induces insulin resistance and predisposes to adverse left ventricular remodeling after pressure overload. Am J Physiol Heart Circ Physiol.

[12] Gerrity RG, Natarajan R, Nadler JL, Kimsey T (2001). Diabetes-induced accelerated atherosclerosis in swine. Diabetes.

[13] Suzuki LA, Poot M, Gerrity RG, Bornfeldt KE (2001). Diabetes accelerates smooth muscle accumulation in lesions of atherosclerosis: lack of direct growth-promoting effects of high glucose levels. Diabetes.

[14] Larsen MO, Wilken M, Gotfredsen CF, Carr RD, Svendsen O, Rolin B (2002). Mild streptozotocin diabetes in the Gottingen minipig. A novel model of moderate insulin deficiency and diabetes. Am J Physiol Endocrinol Metab.

[15] Dufrane D, Gianello P (2012). Macro- or microencapsulation of pig islets to cure type 1 diabetes. World J Gastroenterol.

[16] Larsen MO, Rolin B, Wilken M, Carr RD, Svendsen O (2002). High-fat high-energy feeding impairs fasting glucose and increases fasting insulin levels in the Gottingen minipig: results from a pilot study. Ann N Y Acad Sci.

[17] Dyson MC, Alloosh M, Vuchetich JP, Mokelke EA, Sturek M (2006). Components of metabolic syndrome and coronary artery disease in female Ossabaw swine fed excess atherogenic diet. Comp Med.

[18] Ma YC, Pan YM, Chen L, Chen FM, Yang TT, Chen ML (2014). The research of the insulin resistance atherosclerosis model of mini-swine. Chin J Comp Med.

[19] Chen L, Yang G (2014). PPARs Integrate the Mammalian Clock and Energy Metabolism. PPAR Res.

[20] Tuchscherer M, Kanitz E, Puppe B, Tuchscherer A, Viergutz T (2009). Changes in endocrine and immune responses of neonatal pigs exposed to a psychosocial stressor. Res Vet Sci.

[21] Al-Farai HH, Al-Aboodi I, Al-Sawafi A, Al-Busaidi N, Woodhouse N (2014). Insulin resistance and its correlation with risk factors for developing diabetes mellitus in 100 omani medical students. Sultan Qaboos Univ Med J.

[22] Carroll JA, Daniel JA, Keisler DH, Matteri RL (1999). Non-surgical catheterization of the jugular vein in young pigs. Lab Anim.

[23] Otis CR, Wamhoff BR, Sturek M (2003). Hyperglycemia-induced insulin resistance in diabetic dyslipidemic Yucatan swine. Comp Med.

[24] Christoffersen B, Ribel U, Raun K, Golozoubova V, Pacini G (2009). Evaluation of different methods for assessment of insulin sensitivity in Gottingen minipigs: introduction of a new, simpler method. Am J Physiol Regul Integr Comp Physiol.

[25] Zeng JY, Fang G, Deng M, Zhou L, Liu WQ, Yao F (2014). The anesthesia effect of isoflurane combined with sumianxin II on Tibetan minipigs. Heilongjiang Anim SCI Vet Med.

[26] Seo JP, Son WG, Gang S, Lee I (2011). Sedative and analgesic effects of intravenous xylazine and tramadol on horses. J Vet Sci.

[27] Kuwahara M, Tsujino Y, Tsubone H, Kumagai E, Tsutsumi H, Tanigawa M (2004). Effects of pair housing on diurnal rhythms of heart rate and heart rate variability in miniature swine. Exp Anim.

[28] Canadas L, Ruiz JR, Veiga OL, Gomez-Martinez S, Esteban-Cornejo I, Perez-Llamas F (2014). Obese and Unfit Students Dislike Physical Education in Adolescence: Myth or Truth? The Avena and up&down Studies. Nutr Hosp.

[29] Zhang SF, Chen ML, Chai JG, Pan YM, Ying HZ, Chen L (2009). Effects of salvianolic acid capsule on acute myocardial ischemia in Beagle dogs. Chin J Clin Pharmacol Ther..

[30] Christoffersen B, Golozoubova V, Pacini G, Svendsen O, Raun K (2013). The young Gottingen minipig as a model of childhood and adolescent obesity: influence of diet and gender. Obesity (Silver Spring).

[31] Bender SB, Tune JD, Borbouse L, Long X, Sturek M, Laughlin MH (2009). Altered mechanism of adenosine-induced coronary arteriolar dilation in early-stage metabolic syndrome. Exp Biol Med (Maywood).

[32] Li Z, Woollard JR, Wang S, Korsmo MJ, Ebrahimi B, Grande JP (2011). Increased glomerular filtration rate in early metabolic syndrome is associated with renal adiposity and microvascular proliferation. Am J Physiol Renal Physiol.

[33] Low Wang CC, Lu L, Leitner JW, Sarraf M, Gianani R, Draznin B (2013). Arterial insulin resistance in Yucatan micropigs with diet-induced obesity and metabolic syndrome. J Diabetes Complications.

[34] Fagot-Campagna A, Balkau B, Simon D, Warnet JM, Claude JR, Ducimetiere P (1998). High free fatty acid concentration: an independent risk factor for hypertension in the Paris Prospective Study. Int J Epidemiol.

[35] Egan BM, Hennes MM, Stepniakowski KT, O'Shaughnessy IM, Kissebah AH, Goodfriend TL (1996). Obesity hypertension is related more to insulin's fatty acid than glucose action. Hypertension.

[36] Iimura O, Shoji T, Yoshida S, Sato R, Nohara K, Kudoh Y (1985). Studies on experimental coronary insufficiency. Effect of L-carnitine on myocardial ischemia produced by sympathetic-nerve stimulation with high plasma fatty acids. Adv Myocardiol.

[37] Lopaschuk GD, Ussher JR, Folmes CD, Jaswal JS, Stanley WC (2010). Myocardial fatty acid metabolism in health and disease. Physiol Rev.

[38] Sanchez AA, Singh GK (2014). Early ventricular remodeling and dysfunction in obese children and adolescents. Curr Treat Options Cardiovasc Med.

[39] Gil JS, Drager LF, Guerra-Riccio GM, Mostarda C, Irigoyen MC, Costa-Hong V (2013). The impact of metabolic syndrome on metabolic, pro-inflammatory and prothrombotic markers according to the presence of high blood pressure criterion. Clinics (Sao Paulo).

[40] Mjos OD (1971). Effect of free fatty acids on myocardial function and oxygen consumption in intact dogs. J Clin Invest.

[41] Tiyerili V, Becher UM, Aksoy A, Lütjohann D, Wassmann S, Nickenig G, C.F M. AT1-receptor-deficiency induced atheroprotection in diabetic mice is partially mediated via PPARγ. Cardiovasc Diabetol. 2013;12(30): doi:10.1186/ 1475-2840-1112-1130. 10.1186/1475-2840-12-30 PMC3667017 23374104

[42] Campbell FM, Kozak R, Wagner A, Altarejos JY, Dyck JR, Belke DD (2002). A role for peroxisome proliferator-activated receptor alpha (PPARalpha ) in the control of cardiac malonyl-CoA levels: reduced fatty acid oxidation rates and increased glucose oxidation rates in the hearts of mice lacking PPARalpha are associated with higher concentrations of malonyl-CoA and reduced expression of malonyl-CoA decarboxylase. J Biol Chem.

[43] Asakawa M, Takano H, Nagai T, Uozumi H, Hasegawa H, Kubota N (2002). Peroxisome proliferator-activated receptor gamma plays a critical role in inhibition of cardiac hypertrophy in vitro and in vivo. Circulation.

[44] Maejima Y, Okada H, Haraguchi G, Onai Y, Kosuge H, Suzuki J (2011). Telmisartan, a unique ARB, improves left ventricular remodeling of infarcted heart by activating PPAR gamma. Lab Invest.

[45] Lin Y, Zhu X, McLntee FL, Xiao H, Zhang J, Fu M (2004). Interferon regulatory factor-1 mediates PPARgamma-induced apoptosis in vascular smooth muscle cells. Arterioscler Thromb Vasc Biol.

[46] Blaschke F, Takata Y, Caglayan E, Law RE, Hsueh WA (2006). Obesity, peroxisome proliferator-activated receptor, and atherosclerosis in type 2 diabetes. Arterioscler Thromb Vasc Biol.

[47] Lee H, Shi W, Tontonoz P, Wang S, Subbanagounder G, Hedrick CC (2000). Role for peroxisome proliferator-activated receptor alpha in oxidized phospholipid-induced synthesis of monocyte chemotactic protein-1 and interleukin-8 by endothelial cells. Circ Res.

[48] Cheng L, Ding G, Qin Q, Xiao Y, Woods D, Chen YE (2004). Peroxisome proliferator-activated receptor delta activates fatty acid oxidation in cultured neonatal and adult cardiomyocytes. Biochem Biophys Res Commun.

[49] Marx N, Bourcier T, Sukhova GK, Libby P, Plutzky J (1999). PPARgamma activation in human endothelial cells increases plasminogen activator inhibitor type-1 expression: PPARgamma as a potential mediator in vascular disease. Arterioscler Thromb Vasc Biol.

[50] Lichtenstein O, Safar ME, Mathieu E, Poitevin P, Levy BI (1998). Static and dynamic mechanical properties of the carotid artery from normotensive and hypertensive rats. Hypertension.

[51] Zhang J, Fu M, Zhu X, Xiao Y, Mou Y, Zheng H (2002). Peroxisome proliferator-activated receptor delta is up-regulated during vascular lesion formation and promotes post-confluent cell proliferation in vascular smooth muscle cells. J Biol Chem.

[52] Shchelkunova TA, Morozov IA, Rubtsov PM, Bobryshev YV, Sobenin IA, Orekhov AN (2013). Lipid regulators during atherogenesis: expression of LXR, PPAR, and SREBP mRNA in the human aorta. PLoS One.

